# Bronchopleural fistula in a 5- years old child with novel CARMIL 2 mutation: A rare disease and a rare case

**DOI:** 10.1016/j.amsu.2021.102443

**Published:** 2021-06-06

**Authors:** Ikram ul Haq Chaudhry, Ahmed Alshaer, Burair Al Jassas, Amal Alkhunizi, Mohammad Alsaiary, Tasneem A. AlMubayaedh, Abass A. AlMalki, Ahmed Almesfer

**Affiliations:** Department of Pediatric Thoracic Surgery and Intensive Care Medicine, Infectious Disease, and Immunology, Maternity and Children Hospital, Dammam, Saudi Arabia

**Keywords:** Genetic disease, CARMIL 2 mutation, Respiratory infection, Brochpleural fistula, Surgery

## Abstract

A five year girl had eczema and allergic rhinitis in the past, presented with a history of cough, shortness of breath for the last one month. Her chest -X-ray showed a left side pleural effusion, and a computed tomographic scan (CT) of the chest showed left side hydropneumothorax. Left side 21 Fr drain was inserted. Her clinical condition deteriorated despite antimicrobial therapy, and she required mechanical ventilatory support due to respiratory distress. She also developed a right-sided pneumothorax that was managed by inserting a 21 Fr chest drain. A video-assisted thoracoscopic VATS procedure was done to staple the lung bullae and drain the empyema. Her post-operative chest X-ray showed good lung expansion. Pleural fluid culture report was positive for candida. She was commenced on antifungal microbial therapy. Two days later, she developed again left side pneumothorax, which was again managed by left intercostal drain. We were unable to wean her off from mechanical ventilatory support due to a significant air leak due to bronchopleural fistula. A posterolateral thoracotomy was performed, and the bronchopleural fistula was closed. She was extubated the next day, and intercostal drains were removed on the 4th post-operative day.

## Highlights

• A six year old girl presented with history of eczema, allergic rhinitis and recurrent respiratory tract infections.
She developed left side pneumothorax and given mechanical ventilatory support for two months."
Gene study revealed Rare disease CARMIL2 mutation.
Antifungal, antiviral and antibacterial medications were given.
Immunoglobulin intravenous therapy. Bronchopleural fistula closed with pedicle intercostal muscle.

## 1 Introduction

CARMIL 2 is a multi-domain cytosolic protein essential for cytoskeletal organization cell migration and has a significant role in T-cell signaling. Mutation in CARMIL 2 can lead to immunodeficiency disorder with variable phenotype presentations [1]. This primary immune deficiency in several patients has been reported with pathogenic variants in the capping protein regulator and myosin 1 linker 2 (CARMIL2), also described as RGD leucine-rich repeat tropomodulin and proline-rich -containing protein [2]. These patients can present with different clinical manifestations like recurrent respiratory infections, dermatitis, eczema, psoriasis, esophagitis, diarrhea. Such patients often require multiple hospitalizations due to recurrent infections [3,4]. This case has been reported in line with SCARE criteria [5].

## 2 Case report

**Fig. 1:**
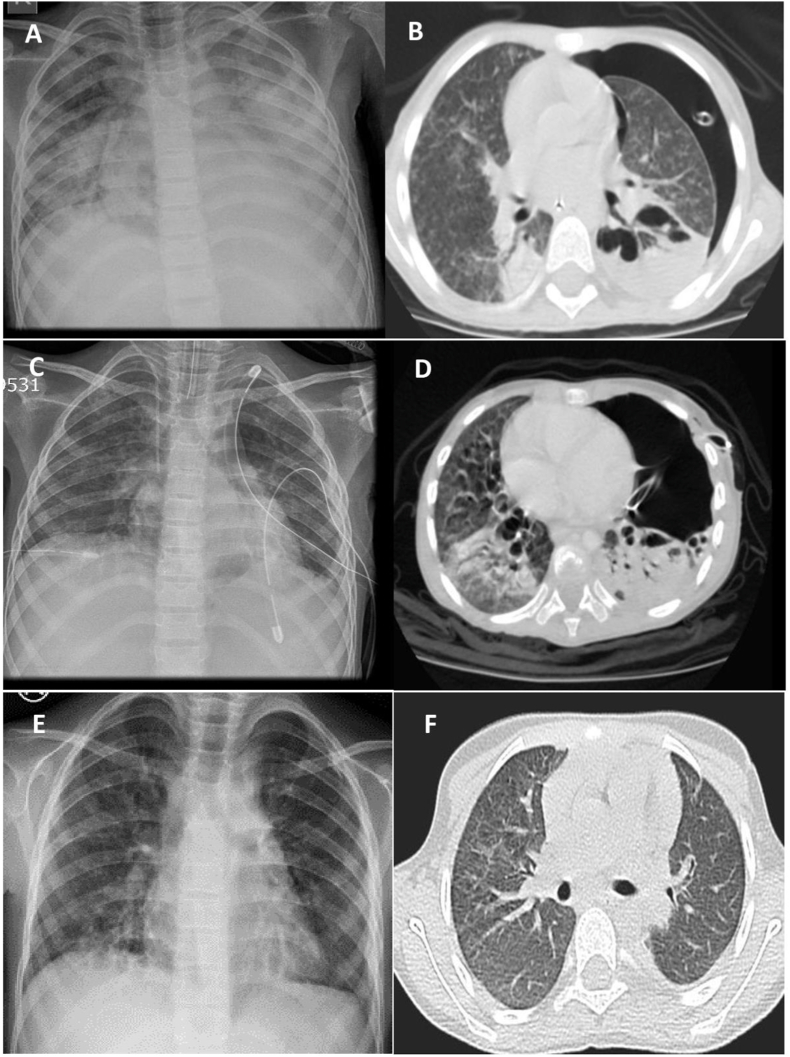
(A) Chest xray showing left side pneumothorax. (B&C) Chest ray &CT scan showed chest drain inserted. (D) Recurent pneumothorax after surgery. (E&F) After closure of Bronchpleural fistula.

A five -year-old girl was admitted with a three-week history of cough and shortness of breath. she has a past history of mild eczema and allergic rhinitis, and recurrent respiratory tract infection. Her chest X-ray showed left-side pneumothorax. CT scan of thorax revealed a left lung Pneumothorax and pleural collection Fig. 1 (A&B). Basic blood investigation showed decreased lymphocyte count. An immunologic evaluation was proceeded due to recurrent respiratory tract infection and persistent low lymphocyte count. Her immunoglobulins levels IgG, IgA were low, and IgM was normal. The lymphocyte subset result showed a decrease in all lines of lymphocytes; the oxidative essay was normal. Gene study (primary immune deficiency panel) revealed a positive for a mutation in the CARMIL 2 gene (c.2536_2548del p. leu846 serf*36. The multidisciplinary team decided to proceed with surgery due to persistent mechanical support and significant air leak. The pediatric surgeon performed VATS stapling of lung bullae and drainage of empyema. Patient clinical condition improved chest X-ray showed lung expansion. Pleural fluid culture for positive for candida Albicans and staphylococcus epidermis, sensitive to Fluconazole and vancomycin, respectively. While the blood culture was positive for Gram-Positive cocci, and PCR was positive for cytomegalovirus. She was commenced on triple therapy antifungal (Fluconazole 240 mg intravenous (I/V) once daily), antimicrobial (Vancomycin 200mg I/V six hourly for seven days, Clindamycin 199 mg I/V six-hourly Antiviral Ganciclovir 100 mg1/V twelve hourly. She also received intravenous immunoglobulin IgG 5% 20 gm stat followed by monthly dose. Two days later, she again developed left pneumothorax, chest drain was inserted. We were unable to wean off mechanical ventilatory support due to a significant prolonged air leak Fig. 1(C&D). A thoracic surgeon was consulted, and he proceeded for surgical closure of bronchopleural fistula through left posterolateral thoracotomy. The fistula was closed with interrupted 3/0 dexon sutures and reinforced with pedicled intercostal muscle flap. The wound was closed in layers, and one chest drain was left in the pleural cavity. Post-operatively there was no further air leak, and we were able to wean her off from the ventilator. The chest drain was removed after four days, and the patient was discharged home after one week for further follow-up in outpatient. Her follow-up chest x-ray and CT scan of the thorax were normal Fig. 1(E and F) She is followed up by internal medicine and immunologist for further care. She continue to receive 5% Immunoglobulin 20 gm on monthly bases.

## 3 Discussion

CARMIL2 deficiency is a genetic disease reported as part of primary immune deficiency in which there is pathogenetic variation in capping protein regulator and myosin one linker two, also called RGD. This is an autosomal recessive disease more prevalent in countries (Tunisia, Morocco, Turkey, Norway, Brazil, Saudi Arabia) where inter-family marriages are common. Patients with CARMIL2 mutation can present with heterogeneous clinical manifestations. Anas et al. reported seven cases of CARMIL2 in Saudi families with clinical manifestation as eczema, dry and scaly dermatitis and recurrent skin abscesses, recurrent respiratory infections, and bronchiectasis [6] Sorte et al. identified four cases of CARMIL2 genetic disease in Norwegian families they presented with phenotype skin warts, dermatitis, and molluscum contagious [7]. Schober et al. also described four cases of CARMIL2 gene mutation presented with Epstein -Barr -virus-related disseminated smooth muscles tumor [8].

Wong et al. described six cases of CARMIL2 mutation in different families [9]. To date, 24 cases have been reported in medical literature Table 1. Mutation in CARMIL2 is also described in association with combined immune deficiency syndrome [10,11]. The patients with disease usually present with recurrent dermatitis, eczema, and dermal abscesses; other clinical manifestations include upper and lower respiratory tract infection, eosinophilic esophagitis, chronic diarrhea (colitis), EBV viremia, food allergy, asthma, and allergic rhinitis, and failure to thrive [12]. The best diagnostic laboratory tool is gene study and Immunophenotyping, which shows a decrease in overall patient regulatory T Cell and low or absent T-cell proliferation upon CD3/CD28 stimulation, skewing towards naïve form [[13], [14], [15]]Early diagnosis is essential to start immunoglobulin and appropriate antimicrobial therapy. The differential diagnosis in such cases is combined immune deficiency syndromes such as CARMIL2,Wiskott-Aldrich and DOCK8 deficiency. All these genetic disease lead to defect in actin formation and are required of TCR signaling (T Cell Receptor) [16].

**Table 1:** Total number of cases of Novel CARMIL2 Mutation to date = 24.

Author	No of Cases	Clinical Manifestation
Sorte et al., 2016	4	Dermatitis, warts, Molluscum contangiosum
Wong et al., 2016	6	Cutaneous and pulmonary, allergy, TB, fungal infection
Schober et al., 2017	4	Epstein -Barr viremia, muscle tumors
Anas et al., 2018	7	Eczema, dry dermatitis, skin abscesses
Alina et al., 2019	1	Infantile colitis
David K et al., 2019	1	Smooth muscle tumors and eosinophilic esophagitis.
chaudhry et al., 2021	1	Dermatitis, respiratory tract infection, and Eczema.Allergic Rhinitis Respiratory tract infection, Bronchopleural fistula

Recurrent respiratory infection can lead to pleural effusion, pneumonia, pneumothorax, and empyema. Bronchopleural fistula (BPF) has never been reported in patients with CARMIL2 disease. Surgical closure of BPF is challenging in such cases due to immune deficiency, fragile tissues, and propensity towards bacterial and fungal infections. Baro trauma due to Mechanical ventilation is another added risk in such cases.

In conclusion, in medical literature, 23 cases of CARMIL2 mutation have been reported to date (Table 1). We report a rare case of immune deficiency CARMIL2 disease in 6 years old girl who had a pneumothorax and recurrent bacterial and fungal respiratory infection. She required prolonged mechanical ventilatory support due to bronchopleural fistula, which has never been reported before. Surgery in such cases is very challenging; we successfully repaired the Bronchopleural fistula using a pedicle intercostal muscle flap. The patient was extubated the next day and discharged home on the fourth post-operative day for further follow-up in outpatient.

## Ethical approval

IRB.

## Source of funding

None.

## Author contribution

Ikram ul Haq Chaudhry Thoracic surgeon, performed second operation drafting article.

Ahmed Alshaer wrote abstract.

Burair Al Jassas Wrote part of case report.

Amal Alkhunizi, Images and references.

Mohammad Alsaiary, intensive care Medicine searches references.

Tasneem A Almubayaedh virologist wrote part of discussion.

Abass A AlMalki paediatric surgeon performed first surgery wrote part of case report.

Ahmed Almesfer consultant infectious disease wrote structured abstract.

## Consent

Yes, written consent was obtained.

## Registration of research studies

1. Name of the registry: Research registry
2. Unique Identifying number or registration( Not requires this is case report)
3. Hyperlink to your specific registration (must be publicly accessible and will be checked): http://www.researchregistry.com/browse-the-registry#home/

## Guarantor

Ikram Chaudhry.

## Provenance and peer review

No commissioned, externally peer reviewed.

## Declaration of competing interest

None.

## References

[1] Kim David, Uner Asyegul, Saglam Arzu, Chadburn Amy, Crane Genevieve M. (2009). Peripheral Eosinophilia in primary immunodeficiencies of Actin dysregulation: a case series of Wiskott -Aldrich syndrome.CARML2 and DOCK8 deficiency and review of the literature. Ann. Diagn. Pathol..

[2] R Roncagalli M., Cuchetti N Jarmuzynski, Gregoire C., Bergot E., Audebert A. (2016). The scaffolding function of PLTRP protein explains the essential role for CD28 costimulation in mouse and human T cells. J. Exp. Med..

[3] Roncagalli R., Cicchetti M., Jarmuzynski N., Gregoire C., Bergot Audebert S. (2016). The scaffolding function of the PLTPR protein explains its essential role for CD 28 costimulation in mouse and human T cells. J. Exp. Med..

[4] Chan A., Bassetti J., Feuille E. (2018). A case of recurrent aspiration pneumonia and persistent opiophilia. Medically challenging causes/Ann Allergy Asthma Immunol.

[5] Agha R.A., Franchi C., Sohrabi C., Mathew G., For the SCARE Group (2020). The scare 2020 Guidelines updating consensus surgical case report(scare) guidelines. Int. J. Surg..

[6] Alazami A.M., Al Helale, Alhissi S., Al Saud B., ALajlan H., Monies D., Zeeshan S., Mohamed A.H., Rand A., Hasan A., Nouf S.A., Farrukh S., Hamoud A. (2018). Novel CARMIL2 mutations in patients with variable clinical dermatitis, infections, and combined immunodeficiency. Front. Immunol..

[7] Sorte H.S., Osens L.T., Fevange B., Aukrest P., Ericchsen H.C., Backe P.H. (2016). A potential founder variant in CARMIL2/PLTPR in three Norwegian families with warts, molluscum contangiosum, and T cell dysfunction. Mol. Genet. Genom. Med..

[8] Schober T., Magg T., Laschinger M., Rolhfs M., Linhares M.D., Puchalka J. (2017). A human immune deficiency syndrome caused by a mutation in CARMIL2. Nat. Commun..

[9] Wong Y., Ma C.S., Ling Y., Bousfiha A., Camcioglu Y., Jacquot S. (2016). Dual T cell -and B cell-intrinsic deficiency in humans with biallelic PLTPR mutations. J. Exp. Med..

[10] Roifman C.M., Somech R., Kavadas F., Nahum A., Dalal I. (2012). I am defining combined immunodeficiency. J. Allergy Clin. Immunol..

[11] Al Herz W., Al Mousa H. (2013). Combined immunodeficiency the middle east experience. J. Allergy Clin. Immunol..

[12] Felgentreff K., Perez- Becker R., Speckmann C., Schwarz K., Kalwak L., Markelj G. (2011). Clinical and immunological manifestations of patients with a typical severe combined immune deficiency. Clin. Immunol..

[13] Matsuzaka Y., Okamoto K., Mabuchi T., Lizuka M., Ozawa A., Oka A. (2004). Identification expression analysis and polymorphism of a novel RLTPR gene encoding RGD motif tropomodulin domain and proline/Leucine-rich regions. Gene.

[14] Lanfer M.H., McConnell P., Cooper J.A. (2016). Cell migration and invadopodia formation require a membrane-binding domain of CARMIL2. J. Biol. Chem..

[15] Maccani Maira E., Speckmann Castren, Heeg Maximilian, Reimer Antonia, Casetti Fedirica, Has Cristina, Ehi Stephan, Castro Carla N. (2019). Profound immune deficiency with severe skin disease is explained by concomitant novel CARMIL2 and PLECI loss of function of mutation. Clin. Immunol..

[16] Williams K.W., Milner J.D., Freeman A.F. (2015). Eosiniphilia associated disorders of immune deficiency or immune dysregulation. Immunol. Allergy Clin. N. Am..

