# Supplementary material for: Bronchopleural fistula in a 5- years old child with novel CARMIL 2 mutation: A rare disease and a rare case
Source: Ann Med Surg (Lond). 2021 Jun 6;66:102443. doi: 10.1016/j.amsu.2021.102443 (PMC8193081; doi:10.1016/j.amsu.2021.102443)
Supplement: Multimedia component 2 [file mmc2.docx]

**Abstract** A five-year girl who had eczema and allergic rhinitis and recurrent respiratory tract infections in the past presented with cough and shortness of breath. Chest and CT scan of thorax showed pneumothorax and pleural fluid. Genetic tests showed she had CARMIL2 mutation a rare immune deficiency syndrome.

**Introduction**

CARMIL2 is a multi-domain cytosolic protein essential for cytoskeletal organization cell migration and has a significant role in T-cell signaling. Mutation in CARMIL2 can lead to immunodeficiency disorder with variable phenotype presentations.1 This primary immune deficiency in several patients has been reported with pathogenic variants in the capping protein regulator and myosin 1 linker 2 (CARMIL2), also described as RGD leucine-rich repeat tropomodulin and proline-rich -containing protein These patients can present with different clinical manifestations like recurrent respiratory infections, dermatitis, eczema, psoriasis, esophagitis, diarrhea. Such patients often require multiple hospitalizations due to recurrent infections. This case has been reported in line with SCARE criteria.

**Case presentation.**

A Five-year-old girl with past history of eczema, allergic rhinitis and recurrent respiratory tract infections presented with recent history of cough and shortness of breath. Her chest –ray and CT scan of thorax showed hydroneumothorax. Blood count showed decreases lymphocyte count and decreased IgA and IgG levels. Gene study (primary immune deficiency panel) revealed a positive for a mutation in the CARMIL2 gene (c.2536_2548del p.leu846 serf*36.She required mechanical ventilation and we were unable to wean her off from mechanical ventilatory support . Due to significant air leak she was operated and brchopleural fistula was closed with pedicle intercostal muscle flap. Post –operative recovery was uneventful.

**Discussion**

. CARMIL2 deficiency is a genetic disease reported as part of primary immune deficiency in which there is pathogenetic variation in capping protein regulator and myosin one linker two, also called RGD. This is an autosomal recessive disease more prevalent in countries (Tunisia, Morocco, Turkey, Norway, Saudi Arabia) Brazil, Saudi Arabia) where inter-family marriages are common. Patients with CARMIL2 mutation can present with heterogeneous clinical manifestations. Recurrent respiratory infection can lead to pleural effusion, pneumonia, pneumothorax, and empyema. Bronchopleural fistula (BPF) has never been reported in patients with CARMIL2 disease. Surgical closure of BPF is challenging in such cases due to immune deficiency, fragile tissues, and propensity towards bacterial and fungal infections. Baro trauma due to Mechanical ventilation is another added risk in such cases.

In conclusion, in medical literature, 23 cases of CARMIL2 mutation have been reported to date.
